# Genes, culture, and scientific racism

**DOI:** 10.1073/pnas.2322874121

**Published:** 2024-11-18

**Authors:** Kevin N. Lala, Marcus W. Feldman

**Affiliations:** ^a^School of Biology, Centre for Biological Diversity, University of St. Andrews, St. Andrews KY16 9TF, United Kingdom; ^b^Department of Biology, Stanford University, Stanford, CA 94305

**Keywords:** gene-culture coevolution, heritability, gene-by-environment interaction

## Abstract

Quantitative studies of cultural evolution and gene-culture coevolution (henceforth “CE” and “GCC”) emerged in the 1970s, in the aftermath of the “race and intelligence quotient (IQ)” and “human sociobiology” debates, as a counter to extreme hereditarian positions. These studies incorporated cultural transmission and its interaction with genetics in contributing to patterns of human variation. Neither CE nor GCC results were consistent with racist claims of ubiquitous genetic differences between socially defined races. We summarize how genetic data refute the notion of racial substructure for human populations and address naive interpretations of race across the biological sciences, including those related to ancestry, health, and intelligence, that help to perpetuate racist ideas. A GCC perspective can refute reductionist and determinist claims while providing a more inclusive multidisciplinary framework in which to interpret human variation.

## The Fallacious Intuitive Argument

Scholars interested in race and ethnicity concur that human races are social constructs, and that there are no meaningful genetic differences between socially defined races (1–5). However, this belief is not held by the general public. Despite decades of public education, surveys show that people still regard race as a biological concept (6–8), a belief linked to racist attitudes (9). That the scientific evidence—so clear to most academics—should fail to convince the public is a serious concern.

We suggest that this failure may be due to “mixed messages” emanating from within and outside academia that undermine the accepted scholarship that race is a social construct. This results in an intuitive but fallacious argument, summarized below:

*People can use skin color and other easily observed anatomical features to allocate individuals to racial categories. These physical differences have a genetic basis, so it is easy to imagine that they are representative of human biology, and that genetic differences between races extend to less-visible characteristics, including temperament and mental ability. In addition, ancestry analyses, confirm that there are genetic differences between populations, while the medical community uses race as a disease-risk criterion, and links differences between populations to the incidence of medical conditions (e.g., stroke, heart disease). The dominance of particular races in some sports, and variation across races in scholastic performance, educational attainment, and IQ, is interpreted as reflecting biological differences. As a result, the assertion that races are social constructs may be regarded with suspicion, and viewed as politically motivated.*

This argument is incorrect but disturbingly persistent and can incite racist actions. With racism and hate crimes on the rise and scientific racism again a concern (10, 11), the situation today recalls that of 50 y ago, when Stanford professors Luca Cavalli-Sforza and Marcus Feldman first began exploring cultural evolution and gene-culture coevolution (CE and GCC). Below we describe how part of the original motivation for this work was to challenge strong hereditarian positions and crude genetic determinism. This 50th-anniversary special edition is an appropriate setting to attempt an authoritative statement on genes, culture, and race. Below we describe the historical context in which CE/GCC emerged, and how the field’s findings can illuminate race-related controversies and are incompatible with racism.

## Eugenics and the Emergence of Gene-Culture Coevolution

In the first half of the 20th century, the orthodox belief among biologists was that most variation in human behavioral traits was due to variation in genes. Eugenics, either “positive” (i.e., increasing the fertility of people with “desirable” traits), or “negative” (reducing the fertility of those with “undesirable” traits) was accepted by mainstream science until the atrocities of Nazi Germany became widely known (10, 12). For instance, it was not until 1954 that the leading journal of human genetic analysis, *Annals of Eugenics*, had its name changed to *Annals of Human Genetics* (12).

During this period the pursuit of eugenics in Europe and the USA was both racist and classist. Of particular relevance to biologists were Hermann Muller’s views on the potential accumulation of deleterious mutations (i.e., “genetic load”) in humans, and the concomitant need to improve the human gene pool (12, 13). The discoveries by Harris (14) and Lewontin and Hubby (15) that human and wild fruit fly populations harbored large numbers of genetic variants at frequencies much higher than those expected of rare deleterious mutations led to a reevaluation of Muller’s load theory, and scientific explanations for the high levels of genetic variation found in natural populations became noneugenic in character. Nevertheless, eugenic accounts of the evolution of human societies have continued to appear in the social science literature.

Pre-World-War-II eugenic pronouncements on the genetic causes of race and class differences in behavior, achievement, and capabilities returned with a vengeance with Berkeley psychology professor Arthur Jensen’s (16) *Harvard Educational Review* article “How much can we boost IQ and scholastic achievement?” Jensen used the high estimated value of the within-population statistic heritability to argue that such a large genetic contribution to measured intelligence made it impossible for interventions by society to reduce the IQ difference between the populations of White and non-White Americans. While Jensen’s thesis appeared in a respected academic journal, the contemporaneous racist speeches by Stanford physicist William Shockley, who openly advocated eugenics, received much wider media attention. Both Jensen and Shockley were roundly criticized by most geneticists. The controversy was swiftly followed by the human sociobiology debate of the 1970s and 80s, and the publication in 1994 of Herrnstein and Murray’s *The Bell Curve*, which reignited the race and IQ debate.

This was the backdrop to the emergence of CE and GCC. Cavalli-Sforza and Feldman were disturbed by the publicity garnered by the writings and speeches of Shockley and Jensen (17), and set out to formulate a better way to compute statistics used to measure familial correlations, based on more-appropriate formal models of coevolutionary dynamics. Their first models showed that parent-to-offspring cultural transmission could produce the appearance of high heritability even though the transmission was not genetic, disproving Jensen’s claim that high heritability implied traits would be difficult to alter (18). Later, they constructed dynamic models incorporating both genetic and cultural inheritance (19–21) and used a variety of methods that extended mathematical evolutionary theory (22, 23). Other researchers, notably anthropologists Robert Boyd and Peter Richerson, contributed to the field’s early development (24, 25) and it has expanded rapidly over the past two decades, introducing a corpus of methodological and conceptual innovations (26–34). While we use the term “gene-culture coevolution,” these studies are also known as “cultural evolution,” “culture-gene coevolution,” and “dual-inheritance theory.”

The best-studied GCC example is the coevolution of milk use with alleles that allow adult humans to digest lactose. Comparative and ancient-DNA analyses reveal that dairy farming appeared before the spread of lactase persistence alleles, generating conditions that made the production of lactase by adults advantageous (35–39). The spread of lactase persistence was shown to depend on the accuracy of cultural transmission (40, 41). However, cultural adaptation to the absence of lactase persistence may also occur, such as the use of milk fermentation by Central Asian herders (42), illustrating the variety of outcomes possible under GCC.

Genomic analyses suggest that gene-culture coevolutionary interactions may be common. Many human genes have been subject to recent positive selection, often in response to culturally transmitted practices (43, 44), which may have created environmental or dietary challenges, eliciting genetic change. For instance, starch consumption is a feature of agricultural societies, but most hunter-gatherers and some pastoralists consume less starch. Humans from different populations have different numbers of copies of the salivary amylase gene (*AMY1*), and copy number correlates positively with the amount of starch in the diet (45). A similar correlation arises with the domestication of rice and associated manufacture of rice wine in different regions of the world and the frequency of a variant of the alcohol dehydrogenase gene (*ADH1B*) that metabolizes alcohol more efficiently, but can make drinking less pleasurable (46). Associations are also found between the frequencies of alleles that protect against a variety of zoonoses or infectious diseases and population density (30, 47). Such studies imply that human activities can modify natural selection thereby affecting inheritance and evolutionary dynamics, a phenomenon known as “niche construction” (48). Relative to other animals, the capacity for niche construction in humans is enhanced through a greater propensity for culture (31, 49–53).

## Gene-Culture Coevolution and Racism

Most GCC research concerns the coevolution of cultural activities that may be associated with panhuman genetic propensities, including individual and social learning, conformity, language, teaching, and various forms of cooperation (24, 30, 54, 55). However, GCC studies have also investigated genetic variation among human populations and its coevolution with culture (e.g., the lactose absorption example). If different cultural activities can elicit genetic responses that differ between different human populations, does that imply that genetic differences between human races are possible? On White supremacist websites, GCC works have been incorrectly claimed to support racist positions.

Interpreting GCC as supporting the existence of biological human races is a serious distortion. For instance, adult lactase persistence alleles reach high frequency in Northern Europe, East Africa, the Middle East, and parts of Asia, while in other regions of these continents, their frequencies are low. This exemplifies a situation of genetic (and cultural) variation within socially defined races, and genetic similarities among populations from different continents (30). The same holds for the copy numbers of amylase genes which differ between different populations of the same socially defined race. Likewise, there are differences between human populations in the incidence of genetic disease that do not map onto racial categories. For instance, sickle-cell anemia is more prevalent in some African populations than others, but also occurs in some Asian countries where malaria is common. In this case, genetic differences exist between culturally defined populations, but are not representative of the wider structure of human genetic variation. Any suggestion that there is a single common culture for each race is also problematic (1).

Contrary to the above, two books on the fringe of GCC appear to affirm the plausibility of human biological races, although neither utilized the models and methods of contemporary GCC, and neither left a legacy on GCC studies. The first is Lumsden and Wilson’s (1981) *Genes, Mind, and Culture* (56), which concluded that significant genetic differences between human societies are likely. Confusion arises because although this work described itself as “gene-culture coevolution” it was actually based on Wilson’s work on human sociobiology. The book’s conclusions are critically dependent on now discredited assumptions, including that transgenerational transmission of culture is weak, and that strong genetic biases influence which cultural traits are acquired. Lumsden and Wilson claimed that strong positive frequency-dependent copying would amplify the effects of modest genetic variation to produce large differences between cultures, a process they labeled “gene-culture amplification.” These claims, which were subject to strong criticism at the time, are inconsistent with extensive data in humans documenting both widespread cultural inheritance (24, 25, 27, 57, 58) and extensive interaction between cognitive domains within the human brain (59, 60), leading to a shift away from claims of “massive modularity” (61). Despite this, Lumsden and Wilson’s “amplification” hypothesis was picked up by journalist Nicholas Wade in his *A Troublesome Inheritance* book to provide a genetic explanation for the strong cultural differences observed between human societies (see ref. 62 for a review).

More recent research has established that human decisions concerning which cultural variants are adopted are not tightly constrained by genes but may change and diversify rapidly, according to a variety of well-documented general learning heuristics, including copying successful or prestigious individuals, or conforming to the majority view (24, 27, 63–66). Cultural transmission allows individuals to adjust to relatively quickly changing aspects of their environments, which would be impossible under tight genetic constraint (24, 25, 67, 68) and leaves Lumsden and Wilson’s primary conclusions untenable (69). A major conclusion of contemporary GCC is that the causal interactions between genes and culture are bidirectional, and that the properties of culture do not reduce to genetic causes.

The second book is Gregory Cochrane and Henry Harpending’s (70) *The 10,000 Year Explosion*, which made provocative claims about a genetic basis of high Jewish intelligence. Eschewing formal GCC theory, these authors claimed that, across Europe, Ashkenazi Jews were forced to be financiers, a cognitively demanding profession in which prosperous individuals would have higher fitness, and these cognitive demands generated selection for genes expressed in high intelligence. Through “back-of-the-envelope-style” calculations they estimated this process to have augmented Ashkenazi intelligence over a 1,000-y period, thus explaining why Ashkenazi Jews today have high average IQ scores. This speculation relies on several contestable assumptions, including low rates of interbreeding between Jews and non-Jews, strong selection, and high heritability of intelligence. The work has the quality of a “just-so story” and, in the context of its racist underpinnings, Cochrane and Harpending’s hypothesis is viewed with strong skepticism by GCC practitioners. There are more plausible explanations for Ashkenazi Jews’ high IQ that involve culturally transmitted norms concerning the importance of education.

## Human Genetics and Race: A Précis

Relative to most other animals, including primates, humans are unusually genetically homogenous (1, 71–73). Fewer than 1 in 1,000 nucleotide bases differ between any randomly chosen pair of humans (72). What genetic variation does exist is not structured into races. Lewontin (74) first reported that the proportion of genetic variation among socially defined human races is low, and suggested that racial taxonomy for our species is biologically meaningless, a conclusion supported by many subsequent studies (e.g., refs. 75–78). Approximately 93% of human genetic variation is found within populations, and only 4% among continents, with the remaining 2 to 3% among populations within continents (76). Most alleles are widely distributed around the world, with few (c. 7%) private to individual continents, and those typically at low frequency; thus there are no distinctive alleles present in all members of one region but absent from individuals outside the region (79). Even for complex traits affected by multiple loci, population genetic theory shows that “*groups are not unduly likely to differ on traits that are determined by many loci, even when the loci influencing the trait would provide a sufficient basis for accurate classification*” (80).

Within evolutionary biology the term “race” has a precise scientific meaning, broadly equivalent to “subspecies,” but human populations are not distinct enough to qualify as races. Subdivision is commonly quantified using a statistic called F_ST_, which relates the genetic variation within and between populations. F_ST_ ranges from 0 (no population subdivision) to 1 (complete subdivision), with an F_ST_ exceeding 0.25 potentially indicating the existence of genetic subgroups (1). An authoritative estimate from the 1000 Genomes Project (81) of the global human F_ST_ was 0.052 to 0.083, revealing that the human population exhibits relatively little genetic subdivision; sharp genetic boundaries and distinct evolutionary lineages of “races” do not exist (1, 82). Rather, human biological diversity is clinal: It changes continuously with geographical separation, as groups merge into each other (83, 84). There may be discontinuities caused by geographic barriers (85), but these barriers are not insurmountable (10). As modern humans migrated from Africa, continuous contact and gene flow meant that they did not form subspecies (1, 86), but rather a “*nested pattern of genetic structure inconsistent with the existence of independently evolving biological races*” (87).

Humans who migrated to higher latitudes experienced a reduction in sunlight, leading to selection that favored lighter skin at several loci (10, 39, 88). However, the distribution and evolution of genes for skin color are not representative of the rest of the human genome, most of which offers little evidence that selection has played a dominant role, with random genetic drift and serial founder effects thought to be more important (79, 89). The genes underlying skin color do not provide a reliable index of genetic differentiation between groups (90, 91).

Edwards (92) claimed that Lewontin (74) had committed a “fallacy” by failing to consider correlations between genes, which he argued would allow individuals to be assigned to racial groups. Subsequent studies have established that large sets of loci can contain a great deal of information about population membership and permit ancestry inferences (76). However, it does not follow that Lewontin’s conclusions about race were fallacious (1, 93). This can be seen by considering the hypothetical case that all genetic variation is geographically continuous and nonclustered; geographical origin is informative but there are no biological races. This scenario is actually a reasonable approximation to reality. Most humans from the same geographic region are fractionally more similar to one another genetically than to individuals from a distant region. However, because of our history of migration and interbreeding, human genetic variation tends to be spatially distributed in a continuous fashion. If a fallacy existed, it was Edwards’ confounding geographical origin with race (17).

Another way to address these issues is to examine what genetic substructure does exist and ask whether it maps onto human races. While the mean genetic difference for two individuals from the same population is almost as large as that for two individuals chosen from any two populations (79), if enough loci are considered, humans can be organized into groups by genetic similarity. Methods exist for partitioning species into clusters of genetically similar individuals, and model-based clustering is a popular technique for quantifying the genetic ancestry of humans and other organisms (Box 1). The methods are generally applied to neutral genetic variation, since most DNA variants that differ in frequency between groups are neutral, functionally insignificant, and probably of little relevance to phenotypic differences between populations (94). For reliable assignment of individuals to continents, over a hundred genetic variants are required, and more for fine-grained population subdivision (91). This reasoning cannot be reversed to predict accurately the genotype of an individual from knowledge of their region of origin; knowing an individual’s continental ancestry only slightly improves the ability to predict their genotype (91).

Statistically estimated clusters using human DNA variants are inconsistent with socially defined races (Box 1). Even those willing, with minimal evidence, to assume that ancestry analyses support the existence of *some* human races, would be forced to conclude that “races” are not those implied by traits such as skin color or eye shape. Ancestry estimated from DNA variants, while itself an ambiguous concept (95), nonetheless provides a far more subtle and complex description of an individual’s genetic makeup than “race” (72, 90). No human populations are “pure” in a genetic sense; individuals do not fall neatly into one of the categories usually defined as “races,” and perceived boundaries between so-called “races” are arbitrary.

Box 1. Findings from cluster analyses inconsistent with human races.

• There is currently no *non-arbitrary* way to decide the number of genetic clusters (i.e., the number *K* of ancestral populations), although there are statistical conventions for estimating *K.*
• There is no single genetic cluster solely associated with “White” people. In the range *K* = 2 to *K* *=* 10, included in the “White” (Eurasian) cluster are some populations from Africa, Central Asia and the Indian sub-continent with dark skin, as well as Islamic and Jewish Middle Eastern populations.
• There is no single genetic cluster solely associated with “Black” people (large regions of Africa are of mixed ancestry and some regions of Africa cluster with non-Africa).
• If *K* is arbitrarily set to 5 to match the most-commonly-specified number of socially defined races, instead of the historically assumed continent-based racial clusters, there are three African clusters, a Eurasian cluster and an Australasian cluster (the latter including Native American and some Asian populations).
• No matter how many clusters there are, almost all humans are of mixed ancestry.

Conclusions based on refs. 10, 81, and 95–100.

## Heritability and Human Inheritance

Heritability is a statistic widely used to estimate the fraction of variation in a phenotypic trait in a population that is due to genetic variation between individuals. The term was introduced in the 1930s as a measure of the sensitivity of agriculturally important traits, such as milk yield or seed production, to selective breeding (102). Known as “narrow-sense heritability” (the fraction of the total phenotypic variance, V_P_, explained by additive genetic variation, V_A_, or *h*^2^ = V_A_/V_P_), this statistic assists the breeder to estimate the likely response to artificial selection. Subsequently, a second definition of heritability emerged from behavior genetics (“broad-sense heritability”, or *H*^2^ = V_G_/V_P_), which estimates the proportion of the phenotypic variation that is genetic (V_G_), including genetic variation that may not be directly responsive to selection, such as allelic dominance (V_D_) or epistatic interactions (V_I_), and variance due to genotype–environment interactions (V_GE_). *H*^2^ has little predictive value, yet it became popular after the 1960s to interpret it as the extent to which the variation in a trait is “genetically determined,” an interpretation that is seriously problematic (103, 104), as we show below.

Two errors in the interpretation of heritability are common (see refs. 105 and 106) for examples; Box 2). The first is to treat heritability as a property of a trait rather than of a sample. Both *h*^2^ and *H*^2^ estimates are highly population specific and cannot be assumed to generalize across populations or environmental conditions. Sample selection can be subject to its own set of biases (107, 108). Most research on human variation relevant to behavior in health has been conducted on WEIRD populations [Western, educated, industrial, rich, democratic; (109), usually in populations of European ancestry [i.e., among the “weirdest” attributes of WEIRD populations may be their whiteness (110)]. Heritability estimates for a trait can differ significantly between human groups, such as between European Americans and African Americans, primarily because those groups are exposed to a different range of environmental conditions. This is also the reason why polygenic scores based on genome-wide association studies (GWAS) of European American phenotypes often perform poorly in predicting variance of the same phenotypes in African Americans (e.g., refs. 111–113).

Box 2. Conclusions about heritability estimates in humans (and other populations with significant extragenetic inheritance).

• If heritability is measured from familial correlations, it is likely to overestimate the proportion of phenotypic variation explained by additive genetic variation.
• If heritability is measured directly from single-nucleotide polymorphisms (SNPs), for multiple reasons, including that extragenetic inheritance contributes to parent–offspring similarity, it is likely to underestimate the extent to which the focal trait is inherited.
• However heritability is measured, its estimation says nothing about the relative importance of genes and environment as causes of the focal character.
• However heritability is measured, its estimation says nothing about the causes of differences between groups in the focal character.
• However heritability is measured, its estimation says nothing about the results of interventions, and is of zero relevance to public policy.

Conclusions based on gene-culture coevolutionary analyses (17, 18, 34, 114–116).

Much of the criticism of Jensen, Shockley, and Herrnstein & Murray centered on their interpretation of the heritability of IQ. Lewontin (117) and Feldman and Lewontin (118) stressed the fallacy of the claim that traits related to IQ, such as educational or economic success, would be impervious to environmental intervention if their heritability was high. Irrespective of its magnitude, heritability is not informative as to the potential impact of interventions. The fact that environmental factors strongly influence IQ is demonstrated by “the Flynn Effect” (119), the finding that across several countries IQ increased markedly from 1947 to 2002 (in the USA it went up by 18 points, a shift that genetic change cannot explain). The Flynn Effect has been attributed to a wide variety of factors, including the educational benefits of movies, television, and computers; improvements in teaching and school resources; preschool education; parenting style; family size; parental education; improved nutrition and health (120). Differences in these factors also provide the most plausible explanation for population differences in IQ, including between European and African Americans (1).

The second mistake is to infer causes of a trait from sources of the variation in that trait. Heritability statistics apportion variation of a trait to genetic and nongenetic sources, but irrespective of the apportionment, they provide no insight into the mechanisms underlying the development of that trait, or of the relative roles that genes and environment might play. This has been illustrated with gene-culture coevolutionary models. Cavalli-Sforza and Feldman (18) first established that vertical (i.e., parent-to-offspring) cultural transmission can inflate genetic heritability estimates for complex traits (see also refs. 17 and 115). Further analyses established that—whether the heritability is estimated from parent–offspring or twin correlations, or on a liability scale, in the presence of vertical cultural transmission—the true heritability (i.e., that based on genetic variation) may depart substantially from such estimates (115). When cultural inheritance is incorporated into statistical models for the determination of phenotypes, and the models are fitted to human familial datasets, sharply lower estimates of the genetic contribution to these phenotypes are obtained (114, 116). For instance, gene-culture coevolutionary modeling reduces estimates of genetic heritability for IQ from standard values of 0.5 to 0.8 when vertical cultural transmission is ignored to 0.29 to 0.42 when it is incorporated; the estimates depend on how cultural transmission is modeled (114, 121, 122). Culture can both mask and unmask genetic variation, leading to decreases or increases in heritability estimates (34). For instance, a substantial rise in the heritability of fertility in the US over the mid-20th century is thought to have been due to an increase in acceptable reproductive choices arising through the diffusion of norms, which unmasked relevant genetic variation (34, 123). In contrast, rigid childbearing norms and the one-child policy in China are thought to have masked genetic variation and reduced heritability estimates (34).

Heritability estimates are also affected by epistasis, gene-by-environment interactions, assortative mating, gene frequencies, and parental construction of the offspring environment (113–115, 124, 125). Thus the same heritability value may be produced by quite different developmental mechanisms. Although references to gene-by-environment interactions are often cursory in GWAS papers, there nonetheless appears to be general recognition that these interactions should be investigated (126). If its primary goal is to decompose the total variance into “nature” and “nurture,” for most human traits the heritability statistic is inadequate. Heritability is a ratio of variances, and cultural transmission can affect both its numerator and denominator. Cultural phenomena are just as capable of generating high heritability estimates as genes (18, 34, 113).

**Fig. 1.**
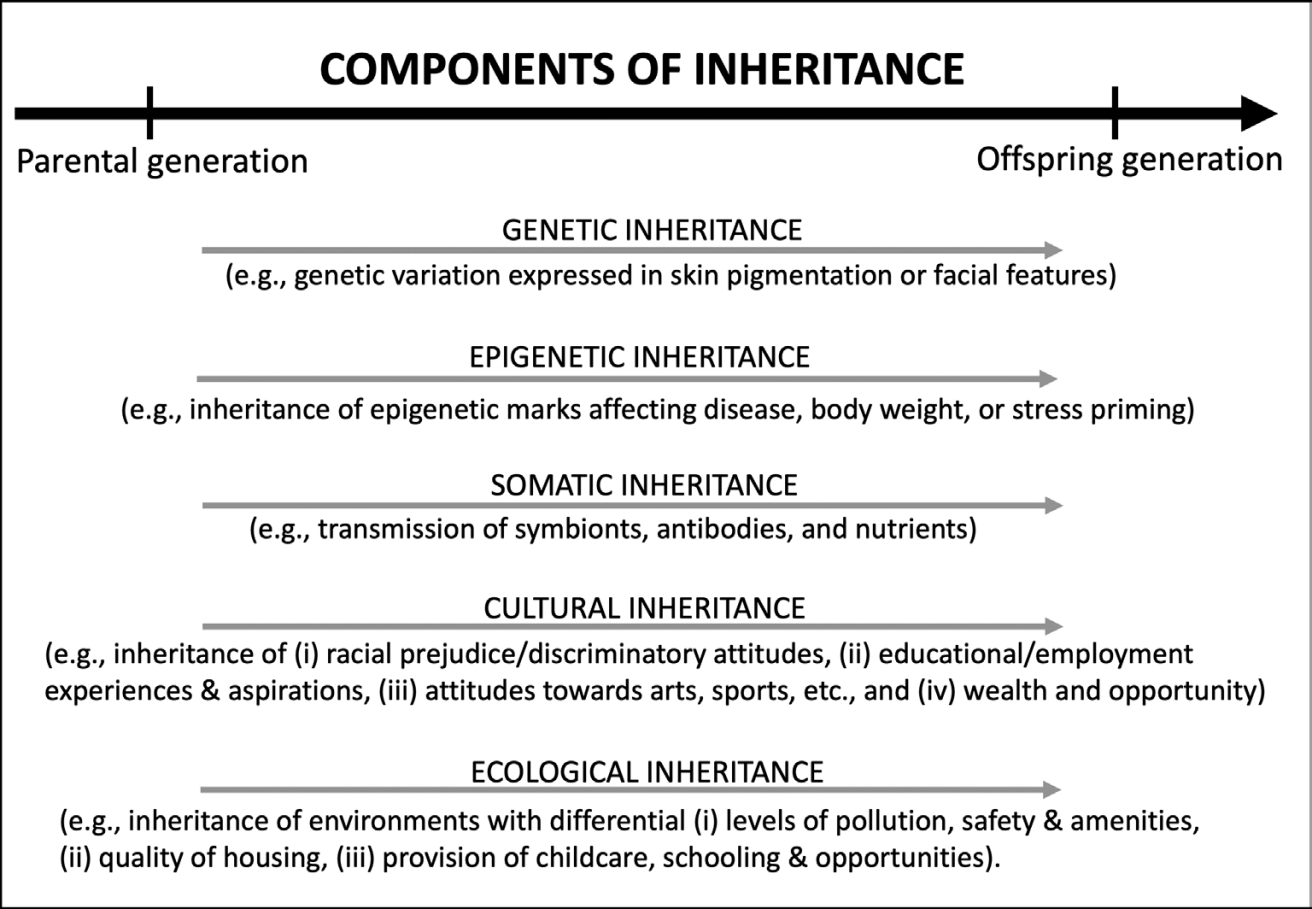
Differences between racialized groups can be affected by several components of inheritance.

When the heritability statistic was devised in the early 20th century, genetic transmission was thought to be the only form of inheritance likely to affect breeding programs. However, recent biological research has led to a multifaceted conception of inheritance, including for humans (Fig. 1). Cultural transmission is not the only form of extragenetic inheritance accounting for differences between individuals and populations. Cellular DNA is clothed in molecules that affect whether specific genes are expressed, and some of these epigenetic phenomena are transmitted across generations (“epigenetic inheritance”) contributing to the inheritance of disease, body weight, stress, aging, and other traits (127). Mammalian babies also inherit some of their mother’s symbiotic bacteria, passed on during childbirth, as well as antibodies in breast milk, and many other nutrients and resources (“somatic inheritance”) (128). They are also born into the environments of their parents; humans inherit birth locations that vary in their quality of housing, schooling, amenities, and pollutants (“ecological inheritance”) (48, 129). Epigenetic, somatic, cultural, and ecological inheritances can affect a wide variety of human traits, and contribute to differences between racialized groups (1, 129). While extragenetic inheritance likely inflates estimates of heritability for many human characters (34, 115, 116) the focus on genetic inheritance has resulted in extragenetic inheritance receiving less attention than it merits. Detailed study of extragenetic inheritance could reveal how differences between racialized groups persist over generations. Inherited experiences, aspirations, attitudes, and wealth combine with epigenetically inherited disease and ecologically inherited environmental conditions to create a “vicious cycle” in which it is easier for the members of some groups to succeed than others, and these differences are likely to be maintained over time. Indeed, extragenetic inheritance is increasingly recognized as a contributor to trait stability (130–132).

For decades, correlations between relatives were the primary tool for estimating the genetic contribution to phenotypic variance. However, in recent years, with the emergence of GWAS, heritability has been estimated by accumulating the phenotypic variance explained by millions of SNPs. SNP heritability is usually lower than estimates based on correlations between relatives, a phenomenon labeled “missing heritability.” Researchers have considered numerous explanations for missing heritability, tending to focus on technological and methodological solutions (116). However, as pointed out by Cavalli-Sforza and Feldman (18), heritability calculated from correlations between relatives can be an overestimate if there is vertical cultural transmission, and other forms of extragenetic inheritance [e.g., epigenetics (133), probably also contribute to this overestimation (116, 124)]. Much of the “missing heritability” might disappear if statistical models took appropriate account of extragenetic inheritance.

## Race and Disease Risk

The false impression that racial categories are biologically meaningful is inadvertently reinforced by the medical community’s use of race as a disease-risk criterion, as well as differences between races in the incidence of medical conditions (e.g., heart disease, sickle-cell anemia). A recent meta-analysis reported that in 59% of clinical trials, self-reported race or skin color was used to identify individuals of African ancestry (82). Responses to medical treatments, such as drugs, are often compared among socially defined races. The medical community’s use of “race” is part of the wider belief that race is a useful means of categorizing people. For instance, forensic databases in the USA are typically organized according to traditional racial categories (72). However, there has recently been a concerted effort by the US National Academies (2, 3) and other groups (4), including the American Association for Physical Anthropology (5), to stress that “race” is not biological. Since human health and well-being can be strongly affected by racism and its societal consequences, this should be noted in the interpretation of GWAS.

Whether or not “race” is an effective predictor of disease risk is a contentious issue but, even if it is predictive, this is unlikely to be because socially defined races differ in disease-relevant genes. First, Europeans carry more genotypes homozygous for putatively deleterious alleles than Africans (134–136) implying that, if genes were the primary explanation, we might expect a greater disease burden in non-Africans. The fact that African Americans commonly have worse health outcomes than European Americans (137, 138) strongly suggests environmental explanations. Second, the overlap between races and genetic ancestry is at best partial (Box 1), and racial categories lack the resolution to be medically informative. Although knowing a patient’s ancestry may be useful in diagnosis and treatment, race will rarely be biologically useful (90, 91). For instance, sickle-cell disease is often described as an African trait, but it is also common in Mediterranean and South Asian populations; it is not a marker of race, but of ancestry from a geographical location where malaria was present (91). Likewise, Tay–Sachs is described as Jewish genetic disease but is also found in French Canadians and the Amish. Here, ancestry, but not race, provides relevant information.

Most health disparities experienced by African Americans are likely to be due to social conditions (1, 82, 129). As Graves and Goodman (1) state: “*the history and current reality of life in a racially stratified and racist society make people sick and shortens their lives.*” This does not mean that ancestry is irrelevant; genetic data can be used to make predictions about susceptibility for some diseases in some populations. However, there is a widespread confounding of the effects of racism and race. In 2004, the Director of the National Human Genome Research Institute wrote: “‘*race’ and ‘ethnicity’ are poorly defined terms that serve as flawed surrogates for multiple environmental and genetic factors in disease causation. Research must move beyond these weak and imperfect proxy relationships*” (see also ref. 82). “Race” is commonly used because it can be identified easily, cheaply, and less invasively, than other data modalities, and because people have been conditioned to think it might be causally relevant, not because it has proven to be a reliable assay of genetic ancestry (94). “Race” is sometimes labeled “self-reported ancestry,” but that label can be misleading. Direct assessment of an individual’s disease-related genetic profile will ultimately yield more accurate and beneficial information than racial or other group membership (72).

Nonetheless, given widespread evidence for social determinants of disease, it is relevant to know whether an individual experiences racism, and race may be a good proxy for that. Socially defined race can become a biological reality when “*cultural constructions of social status, exclusion and exploitation enforced by discriminatory norms, threats and violence”* create *“divergent disease patterns*” (129). Race may be a useful predictor in medicine to the extent that it provides information about social circumstances, lifestyle, and experience of racism, but these socially defined categories should not be confused with biological race (91). In fact, “*social experiences such as being subjected to racism are prime candidates for confounding in genetic studies*” (111). Of the multiple causal factors associated with disease, greater consideration of nongenetic influences is likely to improve outcomes. The polygenic risk scores (PRS) used in GWAS are calculated by summing identified disease “risk” alleles, which are weighted by effect size. It was hoped that the use of PRS would improve health outcomes by accelerating diagnosis and matching patients to treatments. However, participants of European ancestry are overrepresented in GWAS, and the inclusion of participants representing diverse ancestries in research is imperative to ensure equitable benefit from scientific discoveries for all populations, and to prevent further increase in health disparities (111, 139).

## Race, IQ, and Scholastic Attainment

There is no scientific evidence that supports the claims of Shockley, Jensen, Herrnstein, Murray, and other hereditarians that there are substantial genetic differences in intelligence between races. That more than 80% of human genes are expressed in the brain and that humans have a modest global F_ST_ mean that for most genes affecting brain function there cannot be strong genetic differentiation between human populations. In principle, a low global F_ST_ would not preclude a small number of intelligence-related genes of major effect that differed in frequency between socially defined races, but in practice, no such genes have been found. GWAS have consistently failed to discover many variants that are reliably and significantly associated with IQ, and together those few variants predict only 1 to 4% of the variation in IQ test performance (140). Even the most strongly associated variants explain a tiny fraction of variation [e.g., 0.16% for *PLXNB2*, (141)], with no evidence that their incidence varies greatly across socially defined races (1). However, if, as the data suggest, intelligence is affected by many genes of small effect, it becomes implausible that IQ differences between socially defined races arose through a process of random genetic drift; this is relevant because analyses of genetic variation show that recent human evolution has been dominated by drift rather than selection (89). The probability that a long sequence of random changes would all go in the same direction, leading to increases in the intelligence of one population and not others, approaches zero. On the other hand, there is a problem with attributing such differences to natural selection, as there are no compelling hypotheses for why selection would favor greater intelligence in European populations than in other populations (1). Sear (142, 143) shows that recently published claims about average IQ differences between nations are seriously flawed, being reliant on data that fall well below academic standards, yet these data are still regularly used by credulous scholars, leading “to racist conclusions [that] legitimize eugenic arguments.”

Researchers commonly rely on proxies for intelligence, such as educational attainment. The use of such proxies is obviously problematic, as they are clearly influenced by differences, for example in opportunity, wealth, and health. One prominent study identified 74 genetic markers that collectively explain 3.2% of the variance in educational attainment (144). However, a statistical analysis suggested that this association arises partly through genetic variation in fertility rather than intelligence, with female educational attainment negatively associated with having more children (145). These findings highlight that the genetic variants identified by GWAS are not necessarily causal.

The largest GWAS of educational attainment conducted thus far sampled 1.1 million individuals of European ancestry at 1,271 SNPs and reported that polygenic scores explain 11 to 13% of the variation in educational attainment (112). The authors acknowledge that parental effects inflate estimates of the variation explained, stating: “…*some of the predictive power of the polygenic score reflects environmental amplification of the genetic effects. Without controls for this bias, it is therefore inappropriate to interpret the polygenic score for educational attainment as a measure of genetic endowment”* (p. 1116). Such parental effects include vertical cultural transmission, which as noted above, is confounded with genetic effects. The authors continue: “*Because the sample used to construct the score consisted of individuals of European ancestry, we would not expect the predictive power of our score to be as high in other ancestry groups.*” Indeed, when the polygenic score was used to predict educational attainment in a sample of African Americans it could explain only 1.6% of the variance. This difference in GWAS statistics is often attributed to genetic differences between populations, but environmental differences are more likely to explain the differences in such polygenic scores. Diminished educational opportunities for African Americans—including reduced inherited wealth, poorer nutrition, schools, healthcare and amenities, and exposure to harassment and discrimination (1, 120, 146)—reduce variation in educational attainment in this population and affect the sensitivity of GWAS.

Recently, some researchers have attempted to downplay the importance of environmental influences on educational attainment by suggesting that cultural variables are themselves genetically determined. According to this reasoning, cultural differences are driven by genetic differences in parental “quality,” with genetically superior parents producing higher quality offspring both through genetic inheritance and by their superior genes creating better nurturing environments. This “genetification” of culture, sometimes labeled “genetic nurturing” (145), has a sinister side. If all facets of an individual’s life were determined by genetics, rather than social experience and access to resources, then inequities in power and wealth could be (falsely) cast as natural and inevitable (11). Economist Gregory Clark’s (147, 148) suggestion that inheritance of social status in England over a 400-y period is genetic is a case in point. Using a large genealogical database and a variety of status measures, Clark reports that intergenerational correlations in status have not declined over the centuries despite “*vast social changes*” and that the correlations between relatives fit “*a simple model of additive genetic determination of status*” (p. 1). He infers that disparities in status are genetically determined and ineradicable. Such erroneous and dangerous interpretations can result from failure to include cultural transmission in genealogical models, thereby tracing causality only to genes. A more comprehensive view of inheritance is emerging across the biological sciences, and recognizes that the transgenerational stability of traits arises partly through extragenetic inheritance, with parents constructing environments for their offspring. Here, inherited wealth, which was ignored because it does not fit a model of genetic inheritance, is surely a major contributor to the inheritance of social status. The most plausible explanation is that rich people act to retain their wealth and support their families. Clark’s hereditarian assertions have been refuted by Benning et al. (149), who reanalyzed the data and concluded that “*statistical artifacts substantially bias estimates of familial correlations in the paper*” and that “*Clark provides no information about the relative contribution of genetic and non-genetic factors to social status.*” While this study does not mention race, its messages are disturbingly evocative of Jensen’s claims of 50 B.P. and will perhaps inevitably be interpreted similarly.

Indeed, different incarnations of eugenic ideologies keep appearing. Like Jensen (16) and Herrnstein and Murray (150), Ashraf and Galor (151) claimed that differences in historical levels of economic development between different human populations could be explained by differences in the extent of genetic variation among those populations. The economically most successful human populations around the world, it was claimed, had just the right amount of DNA-level variation—not too much, as in Africa, or too little, as in the Americas (see also ref. 152). These claims fit a genetically determinist hypothesis to known facts about human genetic diversity: No evidence exists for a causal relationship between genetic diversity and economic development. Psychology professor Robert Plomin in his book (153) states that parent–offspring correlation in occupational status and income are “*chiefly caused genetically*” (p. 101), that the ability of “*the educational attainment polygenic score … to predict intelligence and reading comes from generalist genes,*” and that “*the most systematic and objective predictor of occupational status and income*” is “*inherited DNA differences*” (pp. 100–101). These claims are reiterated by Harden (154), who states that “*genes cause differences in educational attainment*” (p. 125) and that genetic differences between people “*cause social inequalities … and fertility outcomes like age at first birth*” (p. 129). The intellectual distance between claims of genetic bases for educational attainment, occupational status, and economic success, and eugenic ideas of class and racial differences has never been great. As a result, it behooves scholars to be ready with responses to claims that might initially seem innocuous but may actually turn out to be pernicious.

## The Richer Explanation

Human phenotypic variation is continuously distributed, but because we are a visual species prone to categorical perception, humans have for centuries categorized people into “types,” using the most obvious visual criteria, in particular skin color (30, 155). Modern genetics, however, reveals that we have been misled. The genes underlying skin color are atypical of the human genome, both in their distribution and their evolutionary history, and are not a reliable index of genetic differentiation between groups. There are genetic differences between people, and even modest differences between populations, but genetic variation does not map onto racial categories. Although phenotypic differences exist between socially defined races, such as differences in disease incidence or scholastic performance, these are the product of different life experiences, including racism and discrimination. Such “racial” differences can persist over time, but not because of genetic transmission, which is now recognized to be just one component of human inheritance. Rather, humans have constructed “inequitable niches” (129) which persist through the legacies of inherited norms and institutions, inherited wealth and power, inherited values and traditions, and inherited environments that vary in their amenities and opportunities. These inequitable niches explain why Ashkenazi Jews score highly for IQ, why Jamaicans excel at sprinting, and why African Americans are more likely to die of heart disease. That these and other discrepancies between socially defined races can be attributed to genetic differences, may be intuitive, but it is wrong. Recognition of the importance of extragenetic inheritance to human traits is vital to prevent a return to discriminatory policies falsely premised on the genetic determination of complex human phenotypes. Natural and social scientists have an important role to play in disseminating this multifaceted explanation for racial disparities to the wider public (1–6, 129) By helping to partition variation more appropriately, and by explicitly recognizing the multiplicity of interacting causal paths, analyses that incorporate CE and GCC can complement other excellent resources (1, 11, 129, 155, 156) as important tools to this end.

## Data Availability

There are no data underlying this work.
